# Ultrasound in legal medicine—a missed opportunity or simply too late? A narrative review of ultrasonic applications in forensic contexts

**DOI:** 10.1007/s00414-021-02661-5

**Published:** 2021-07-22

**Authors:** Dustin Möbius, Antonia Fitzek, Niels Hammer, Axel Heinemann, Alexandra Ron, Julia Schädler, Johann Zwirner, Benjamin Ondruschka

**Affiliations:** 1 Institute of Legal Medicine, University Medical Center Hamburg-Eppendorf, Hamburg, Germany; 2 Institute of Macroscopic and Clinical Anatomy, University of Graz, Graz, Austria; 3 Department of Orthopedic and Trauma Surgery, University of Leipzig, Leipzig, Germany; 4 Fraunhofer IWU, Dresden, Germany; 5 Department of Anatomy, University of Otago, Dunedin, New Zealand

**Keywords:** Minimally invasive autopsy, Postmortem imaging, Study review, Ultrasound

## Abstract

**Objectives:**

Conventional autopsies remain the gold standard of postmortem healthcare quality assurance and help gathering extended knowledge on diseases. In answer to constantly declining autopsy rates non- or minimally invasive autopsy methods were introduced. Ultrasound is a well-established tool for imaging commonly used in clinical practice. This narrative review aims to summarize the current literature regarding the feasibility and validity of ultrasound in a forensic context.

**Material and methods:**

A PubMed database search was carried out. Abstracts were scanned for pre-defined ex- and inclusion criteria, followed by a snowball search procedure applied to the primarily included articles.

**Results:**

Forty-five publications met our inclusion criteria. The selected articles concern the feasibility of ultrasound in pre- or postmortem settings, forensic age estimation, and minimally invasive approaches. For imaging, ultrasound was deemed a reliable tool for the examination of epiphyses und superficial wounds, with limitations regarding internal organs and image quality due to postmortem changes. Ultrasound-guided minimally invasive approaches yielded higher success rates for adequate tissue sampling. Many investigations were carried out in low- and middle-income countries focusing on infectious diseases.

**Conclusion:**

Ultrasound seems a promising but underutilized imaging tool in legal medicine to date. Promising approaches on its feasibility have been conducted. Especially for minimally invasive methods, ultrasound offered significant improvements on qualified biopsy sampling and thus appropriate diagnostics. Moreover, ultrasonic evaluation of epiphyses for age estimation offered valuable results. Nevertheless, further assessment of ultrasonic feasibility in forensic contexts is needed.

## Introduction

Ultrasound is a commonly used tool in clinical settings worldwide. Despite its striking clinical benefits, broad availability, and ease of use, ultrasound is to date only sparely used in a postmortem setting. Postmortem ultrasound (pmUS) provides the opportunity to detect a broad range of pathologies such as pericardial tamponade, metastasis, or free abdominal fluid. These findings aid forensic pathologists assessing potentially lethal diagnoses even prior to autopsy or offering valuable information to initiate further investigations on the body [1–6]. Nevertheless, over the last decades, only a handful of studies were published using or focusing on ultrasound as a tool to complement forensic investigation. In times of digital medicine offering the possibility to use highly resolving devices to examine corpses and tissues such as computed tomography (CT) or magnetic resonance imaging (MRI), less sophisticated imaging tools appear to diminish. Regarding the latter, it comes without saying that only a small fraction of the world’s population is equipped with costly devices compared to low- and middle-income-countries (LMICs) [7, 8]. These significant differences in resource availability emphasize the need for affordable diagnostic tools. In the past years, non-invasive or minimally invasive autopsy techniques (MIA) were on the rise both in high-income countries (HICs) as well as in LMICs for different reasons [9].

First, decreasing autopsy rates are reported worldwide, though discrepancy continues to persist between clinical and autopsy diagnoses [10–12]. Common reasons for this trend are religious implications, reluctance of legal guardians to agree to an autopsy due to the inevitable mutilation of the body as a consequence of the necropsy, or simply due to limited ambitions of the clinician. Non- or minimally invasive autopsies could potentially gain higher acceptance still providing the needed postmortem data [13].

Secondly, in LMICs where medical structures fail permitting routine postmortem investigations, or people’s acceptance to conventional autopsy (CA) is low, less invasive methods offer the possibility to reduce the uncertainty about specific causes of death, thereby improving public healthcare systems [14–17].

Also, in those cases with highly contagious diseases, the protection of medical personnel is essential. In consequence, less-invasive procedures minimizing risk to self-injury seem attractive. Especially, in times of global pandemics such as the current COVID-19 pandemic, when there is an urgent demand to understand emerging pathologies, diagnostic postmortem methods have to be safe and widely available. Apart from postmortem diagnostics, ultrasound plays an increasing role in examining children and adolescents in forensic age estimation aiming to reduce the application of radiation and to ease up the investigation procedure [18, 19]. Although advantages of using ultrasound were documented in several publications within the last years, to the best of our knowledge, no summary of its forensic applications and diagnostic benefits is currently available.

Therefore, the following aims were defined for this review:

- to provide a compilation and assessment of studies using ultrasound in the field of legal medicine
- to discuss potential advantages and disadvantages of the method
- to point out potential applications for future research

## Material and methods

### Database search and article selection

A PubMed database search was conducted in April 2020 and then repeated in January 2021 including all relevant results published until December 2020 inclusive. There were no limitations set on the year of publication. A combination of the following key words and related terms was used for the search: “ultrasound,” “sonography,” “legal medicine,” and “minimally invasive autopsy.” The following inclusion criteria for publications were applied:

- ultrasonic investigations of subjects alive or deceased to describe or detect pathological changes
- age estimation by evaluating bones and their epiphyseal fusions by means of ultrasound
- tissue sampling in minimally invasive autopsies supported by ultrasound
- case reports in forensic cases where ultrasound offered essential information

**Fig. 1:**
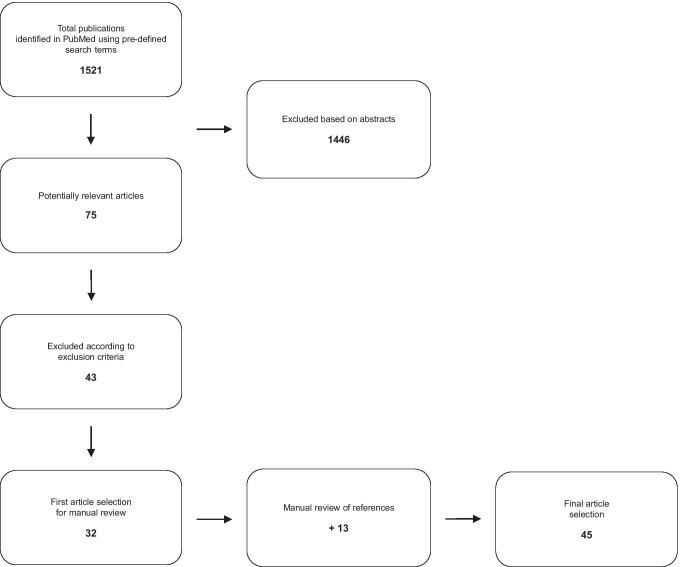
PRISMA flowchart of article selection

Only texts in English or German were selected. Letters or commentaries were excluded. Studies on perinatal and neonatal deaths (babies were born alive but died before 28 days of age), animals, or investigations focusing on reconstruction aspects, e.g., facial tissue measuring using ultrasonic waves, were excluded as well. The results gained by the database research were reviewed manually by one investigator (DM). Duplicates were removed, and titles and abstracts were scanned for ex- and inclusion criteria. Meeting the latter, a review of the full publication was performed, thus creating a start set of publications. Following this, a review of references was conducted applying a snowballing procedure [20] until no more new references could be detected (see PRISMA workflow in Fig. 1).

## Results

A total of 45 publications were found and subsequently included in this review (Fig. 1). The year of publication ranged from 1986 to 2020. Thirteen articles refer to the use of ultrasound in alive patients regarding examination of injuries, or refer to pmUS applicability (Table 1). Eighteen articles were retrieved, focusing on minimally invasive autopsies and its partly support by the use of ultrasound (Table 2). In this group, there were six publications in which the use of ultrasound is not described explicitly [33–37, 39]. All these authors refer methodically to Castillo et al. who described the use of ultrasound for organ detection prior to puncture and did not mention other imaging methods instead of pmUS [38]. For this reason, the studies mentioned above were also included. Fourteen articles were found, in which ultrasound was used for forensic age estimation (Table 3).

**Table 1:** Studies using (postmortem) ultrasound in legal medicine; article details, study population, and main results listed in a chronologically descending order

Author	Year	Journal	N (m/f)	Age range (y)	Time (h) to		Method	Ultrasound device	Strategy of US	Key points
					(pm)US	CA				
Ichioka et al. [21]	2020	Diagnostics (Basel)	402 (259/143)	n.g	n.g	n.g	Determination of bone mineral density of the calcaneus	Benus α (Ishikawa Seisakusho LTD)	Calculation	No correlation between bone mineral density and PMI
Helm et al. [22]	2016	Forensic Science International	18 (n.g.)	1–95	3–717	x	Ultrasound of bruises	n.g	Ultrasonic evaluation (focused)	Additional value for depth and subcutaneous size of bruises
Charlier et al. [23]	2013	Medicine, Science and the Law	38 (28/10)	15–81	< 48	< 48	pmUS vs. CA	ACUSON 128XP/10 (Siemens)	Ultrasonic evaluation	Restrictions based on putrefaction; no lethal diagnoses were detected sonographically
Mimasaka et al. [24]	2012	Legal Medicine (Tokyo)	15 (11/4)	1–76	3–87	n.g	pmUS of bruises in children and deceased	SonoSite180PLUS (Sonosite)	Ultrasonic evaluation (focused)	Sonographic, macroscopic and microscopic determination of subcutaneous hemorrhage thickness
Buthani et al. [25]	2009	Pancreas	17 (17/0)	53–87	n.g	n.g	EUS of excised pancreata and histology	GFUM30 (Olympus America)	Ultrasonic evaluation	Sensitive detection of soft tissue alterations
Seifert & Püschel [26]	2006	Forensic Science International	1 (1/0)	3.5	n.g	x	Ultrasound of the forehead	n.g	Ultrasonic evaluation (focused)	Detection of an extensive subgaleal hematoma in a case of child abuse
Uchigasaki et al. [5]	2006	Forensic Science International	534 (n.g.)	n.g	n.g	n.g	pmUS of pericardium	Sonosite 180II and Sonosite 180Plus (Sonosite)	Ultrasonic evaluation (focused)	Comparison to CA, 11/13 cases of pericardial tamponade could be detected
Uchigasaki et al. [4]	2004	Forensic Science International	158 (108/49)	59:5 ± 19:3	< 192	x	pmUS	Sonosite180 (Sonosite)	Ultrasonic evaluation	Detection of forensically relevant findings in 25%
Uchigasaki et al. [3]	2003	Rechtsmedizin	20 (17/3)	61.3 ± 10.1	n.g	x	pmUS for urine volume determination	Sonosite180 and Sonosite180plus (Sonosite)	Ultrasonic evaluation (focused)	Good feasibility of urine volume determination
Quan et al. [1]	2003	Legal Medicine	85 (63/22)	1–90	7–72	7–72	pmUS for densitometry of lung tissue	LOGIC α200 (GE Yokogawa Medical Systems)	Calculation	High US density is connected to pulmonary edema
Cotton et al. [27]	2000	Echocardiography	1 (1/0)	36	n.g	x	Echocardiography after penetrating chest trauma and pericardiocentesis	n.g	Ultrasonic evaluation (focused)	Forensic echocardiography excluded iatrogenic ventricular injury
Gniadecka et al. [28]	1995	Acta Dermato-Venereologica	1 (1/0)	40		x	Ultrasound of superficial wound	Dermaflex (Cortex Technology)	Ultrasonic evaluation (focused)	Description of scar depth
Webb et al. [2]	1986	Forensic Science Society	5 (n.g.)	n.g	< 20	x	Ultrasound velocity for time of death determination	Smith-Kline ultrasound scanner	Calculation	Prediction of PMI, mean error 5.5%

Legend: x = not performed; *CA*, conventional autopsy; *EUS*, endoscopic ultrasound; *h*, hours; *m/f*, biological sex male/female; *n.g.*, not given; *PMI*, postmortem interval; *(pm)US*, (postmortem) ultrasound; *y*, years

**Table 2:** Minimally invasive autopsies supported by ultrasound; article details, study population, and main results listed in chronologically descending order

Author	Year	Journal	N (m/f)	Age range (y)	PMI (h) to		Method	Ultrasound device	Cause of death Determination			Strategy of US	Key points
					MIA	CA			MIA	CA	Overall concordance		
Li et al. [29]	2020	Histopathology	30 (20/10)	39–91	< 2	x	MIA (COVID-19)	EPIQ 7C (Philips Medical Systems)/Mindray portable Ultrasound M9 (GE Healthcare)	n.g	x	n.g	Ultrasound-guided biopsies	Effective method, 28/30 showed diffuse alveolar damage, fibrosing pattern linked to ventilation time
Brook et al. [30]	2020	Abdominal Radiology	5 (2/3)	58–91	< 3	x	MIA (COVID-19)	iU22 (Philips Medical Systems)	n.g	n.g	n.g	Ultrasound-guided biopsies	Determination of viral loads in obtained tissues
Duarte-Neto et al. [31]	2020	Histopathology	10 (5/5)	33–83	n.g	x	MIA (COVID-19)	Sonosite M-Turbo R (Fujifilm)	n.g	x	n.g	Ultrasound-guided biopsies	Effective method for tissue sampling, no comparison to conventional autopsy
Duarte-Neto et al. [32]	2019	PLOS Neglected Tropical Diseases	20 (15/5)	24–86	n.g	n.g	MIA & CA (Yellow fever)	Sonosite M-Turbo R (Fujifilm)	100.0%	100.0%	100.0%	Ultrasound-guided biopsies and evaluation	Investigation was focused on yellow fever
Palhares et al. [33]	2019	Virchows Archiv	61 (39/22)	14–82	< 48	< 48	MIA & CA, pathological and microbiological Investigation	n.g	100.0%	100.0%	90% (Kappa = 0.777; 95% CI 0.608–0.946)	Partly ultrasonic evaluation, biopsies mostly without US	61% were HIV-positive, infectious diseases representing over 70% of deaths
Hurtado et al. [34]	2018	Scientific Reports	223 (*)	n.g	< 24 vs. > 24 (4–65)	< 24 vs. > 24 (4–65)	MIA and CA, early and late autopsy	n.g	n.g	99.5%	62 to 89% (Kappa = 0.374 to 0.8372; CI: n.g.)	Partly ultrasonic evaluation, biopsies mostly without US	No significant difference could be detected, raising number of bacteria with increasing PMI
Bassat et al. [35]	2017	PLOS Medicine	54 (37/17)	≥ 1 months–15	4–56	4–56	MIA and CA of pediatric deaths	n.g	96.0%	100.0%	89% (Kappa = 0.70; 95% CI 0.49–0.92)	Partly ultrasonic evaluation, biopsies mostly without US	Histopathological and microbiological investigations
Castillo et al. [36]	2017	PLOS Medicine	57 (0/57)	15–39	< 24–64	< 24–64	MIA and CA of maternal deaths	n.g	84.0%	98.0%	68% (Kappa = 0.48; 95% CI: 0.31–0.66)	Partly ultrasonic evaluation, biopsies mostly without US	Disseminated infections
Castillo et al. [37]	2016	PLOS Medicine	112 (57/55)	16–76	< 24	< 24	MIA and CA, pathological and microbiological investigation	n.g	89.2%	100.0%	75.9% (Kappa = 0.732, 95% CI: 0.615–0.838)	Partly ultrasonic evaluation, biopsies mostly without US	Disseminated infections, 59.8% HIV-infected, very low diagnostic rate for cardiovascular diseases in MIA
Castillo et al. [38]	2015	PLOS One	30 (12/18)	17–76	< 24	x	MIA and CA, pathological and microbiological investigation	Mindray portable ultrasound Z6 (Mindray Med Int. Ltd.)	83.0%	n.g	n.g	Partly ultrasonic evaluation, biopsies mostly without US	Leading cause of death were infectious diseases
Martìnez et al. [39]	2015	Diagnostic Microbiology and Infectious Disease	30 (12/18)	17–76	< 24	< 24	MIA and CA, pathological and microbiological investigation	n.g	83.4%	n.g	n.g	Partly ultrasonic evaluation, biopsies mostly without US	Infectious cause of death in 46.7%, etiological agent was detected in 89%
Cox et al. [40]	2014	Journal of Acquired Immune Deficiency Syndromes	96 (45/51)	IQR 29–40	IQR 4–12	IQR 4–12	MIA and CA of HIV-positive patients	Portable ultrasound Vscan V1.2 (GE Healthcare)	n.g	n.g	52% (95% CI: 42–61)	Ultrasound-guided biopsies	Focused on HIV-infected patients, no increase in concordance, using ultrasound vs. blind biopsies, false-positive results due to missing macroscopic aspects
Cox et al. [41]	2014	BMC Clinical Pathology	96 (45/51)	IQR 29–40	IQR 4–12	IQR 4–12	MIA of HIV-positive patients	Portable ultrasound Vscan V1.2 (GE Healthcare)	x	x	x	Ultrasound-guided biopsies	Higher success rate for heart and kidney using ultrasound compared to blind biopsies
Denzer et al. [42]	2013	Gastrointestinal Endoscopy	8 (4/4)	57–92	6–89	5–89	MIA using EGD and EUS	Echoendoscope (Olympus Corp)	n.g	n.g	n.g	Ultrasonic evaluation, few fine needle aspirations	Advantages of macroscopic aspects, higher acceptance compared to CA, EUS was only performed in 8/20, CA in 6/20, only minor diagnoses were detected
Wong et al. [43]	2012	PLOS One	39 (20/19)	IQR 32–40	5–55	x	MIA, pathological and microbiological investigation of HIV-positive patients	n.g	n.g	x	x	Ultrasound-guided biopsies (partly)	69% died due to tuberculosis, high success rate for US-guided biopsies
Weustink et al. [44]	2009	Radiology	30 (19/11)	46–79	4–16	13–18	CT, MRI und US-guided biopsies	n.g	n.g	n.g	77% (n.g.)	Ultrasound-guided biopsies (partly)	Very good agreement between MIA and CA, exception for cardiac diseases, high success rate for US-guided biopsies
Fariña et al. [45]	2002	Virchows Archiv	100 (n.g.)	n.g	n.g	n.g	pmUS, MIA, CA	Sonoline SI-250 (Siemens)	n.g	n.g	83% (n.g.)	Ultrasonic evaluation and ultrasound-guided biopsies	High concordance rate, limited accuracy for cardiovascular and tracheobronchial tree
Fariña et al. [46]	1998	Journal d ‘Echographie et de Médecine par Ultrasons	130 (n.g.)	n.g	n.g	n.g	pmUS, MIA, CA	Sonoline SI-250 (Siemens)	n.g	n.g	92.3%, (95% CI: 86–96)	Ultrasonic evaluation and ultrasound-guided biopsies	High concordance rate, ultrasonic evaluation combined with biopsies

Legend: x = not performed; * = children (54), women in childbearing age (57), other adults (112); *CA*, conventional autopsy; *CI*, confidence interval; *CT*, computed tomography; *EGD*, esophagogastroduodenoscopy; *EUS*, endoscopic ultrasound; *IQR*, interquartile range; *HIV*, human immunodeficiency virus; *h*, hours; *m/f*, biological sex male/female; *MIA*, minimally invasive autopsy; *MRI*, magnetic resonance imaging; *n.g.*, not given; *(pm)US*, (postmortem) ultrasound; *y*, years

**Table 3:** Forensic age estimation; article details, study population, and main results listed in chronologically descending order

Author	Year	Journal	N (m/f)	Age range (y)	Method	Stages of ossification	Key points
Herrmann et al. [47]	2020	European Radiology	33 (33/0)	14–19	Sonography and MRI of the epiphyses of the right femur, tibia, and fibula	3	Clear age separation is possible, individual is younger than 18 years when stage 1 or 2 is seen; good correlation between both imaging methods
Benito et al. [48]	2018	Forensic Science International	221 (75/146)	5–30	Sonography of the medial clavicle epiphysis	4	Overlapping among age groups, stages 3 and 4 estimate the age over 18 years in both sexes
Gonsior et al. [49]	2016	International Journal of Legal Medicine	410 (195/215)	14–26	Sonography of the medial clavicle epiphysis using high-frequency ultrasound	4	Stage 4 could be detected in a considerable number younger than 18 years, no reliable determination of age 18 or 21
Sánchez et al. [50]	2016	International Journal of Legal Medicine	221 (75/146)	5–30	Sonography of the proximal humeral epiphysis	6	Full fusion appears in subjects over 17 years, stage 4 means under 16 years (males) and 15 years (females)
Schmidt et al. [51]	2014	Rechtsmedizin	616 (309/307)	10–25	Sonography of the trochanter major femoris	4	Clear-cut classification in 613 subjects, occurrence of stage 4 at least at 13.5 in females and 14.5 in males
Schulz et al. [52]	2014	Journal of Forensic and Legal Medicine	616 (309/307)	10–25	Sonography of the olecranon	4	Clear-cut classification was possible, ossification stage 3 at the age of 10 years (females) and 13 years (males)
Gonsior et al. [53]	2013	International Journal of Legal Medicine	5 (5/0)	16–29	Sonography and CT of the medial clavicle epiphysis in male cadavers	4	CT remains gold standard, discrepancies in 7/10 cases, overestimation using sonography
Schmidt et al. [54]	2013	International Journal of Legal Medicine	615 (309/306)	10–25	Sonography of the distal radius	4	Clear-cut classification was possible, occurrence of stage 4 at least 15.0 (females) and 15.2 years (males), no prediction on completion of the 18th year possible
Schmidt et al. [55]	2013	Science & Justice	616 (309/307)	10–25	Sonography of the iliac crest	4	Occurrence of stage 4 at 14.4 in males and 17.9 in females at the earliest
Schulz et al. [56]	2013	Forensic Science Medicine and Pathology	616 (309/307)	10–25	Sonography of the medial clavicle epiphysis	4	Stage 4 proofs that an individual has reached at least an age of 18 years
Schulz et al. [57]	2013	Archiv für Kriminologie	616 (309/307)	10–25	Sonography of the distal fibula epiphysis	4	Ossification stage 4 means completion of age 14 in males, 13 in females
Schmidt et al. [58]	2011	International Journal of Legal Medicine	39 (23/16)	11–22	Sonography of the iliac crest	4	Clear-cut classification was possible, occurrence of stage 4 at least 18 years in males and 17.1 in females
Quirmbach et al. [59]	2009	International Journal of Legal Medicine	77 (77/0)	18–24	Sonography of the medial clavicle epiphysis	4	Clear-cut classification was possible, false-positive assessment in 6 of 35 cases, which had not yet reached age 21
Schulz et al. [19]	2008	International Journal of Legal Medicine	84 (45/39)	12–30	Sonography of the medial clavicle epiphysis	4	Classification was possible in 80/84, earliest age of occurrence of stage 4 was 22.9 (males) and 22.5 (females)

Legend: *CT*, computed tomography; *MRI*, magnetic resonance imaging; *m/f*, biological sex male/female; *y*, years

### Experiences of forensic ultrasound

Five articles, published between 1995 and 2016, describe the examination of wounded individuals by means of ultrasound. Mimasaka et al. and Helm et al. used ultrasound to depict and measure subcutaneous hemorrhages showing additional values for visualization of depth and thickness of the bruises beyond the macroscopic aspects [22, 24]. As a case report concerning child abuse, Seifert and Püschel could detect a subgaleal hematoma sonographically in a child presenting a palpable doughy swelling of the forehead which has not been detected by x-ray [26]. In the case reports presented by Cotton et al. and Gniadecka et al., ultrasound was used to examine patients’ injuries. In one patient who underwent pericardiocentesis following a penetrating chest trauma, a verified traumatic ventricular septal defect could be proofed to be caused by the primary penetration and not iatrogenic by reconstructing the entry canals [27]. In one case of a male applying for refugee status, ultrasonic examination of a scar remaining after sharp violence following torture which appeared superficially ultrasound could show a deeper expansion of the scar, thereby providing proof credibility of the statement [28].

Further to this, in a few studies published between 1986 and 2020, ultrasound was used to investigate postmortem tissue. Webb et al. examined undissected cadaveric muscles, measuring the velocity of ultrasound waves corrected to a steady temperature and were able to calculate the postmortem interval (PMI) with an error range of 5.5% within the known PMI [2]. However, this method failed implementation in a daily forensic routine and was discontinued being deployed for this purpose as can be derived from existing literature. Similarly, Ichioka et al. determined the bone mineral density of the right calcaneus of deceased to calculate the PMI, but could not find a correlation between these values [21]. To the best of our knowledge, these were the only past investigations applying ultrasound for time since death determination.

Quan et al. determined ultrasound density of exenterated lungs showing no correlation to PMI, and high-density values in lungs weighing over 1400 g, when measured combined. A contribution of pulmonary edema and ultrasound density was suspected [1]. Another examination was performed by Bhutani et al., who placed pancreata in a vessel filled with fluid medium and examined them by means of endoscopic ultrasound (EUS). Sono-morphological features were determined followed by histopathological evaluation showing positive correlation between both diagnostic methods [25]. Five articles, published between 2003 and 2013, examined complete cadavers by means of ultrasound partly followed by dissection [3–6, 23]. Summarized, Uchigasaki et al. investigated 712 cadavers including cases of natural and non-natural deaths. On one hand, they focused on the feasibility to depict local fluids like urine or pericardial effusions/tamponades and other pathologies. On the other hand, they partly compared their results to CA, which was the first reported approach combining diagnostic sensitivity and specificity of pmUS and CA. In 2003, they examined 158 cadavers, and in about 25% of cases, pathological relevant findings could be detected sonographically [3]. In 2006, a total of 534 corpses were investigated focusing on the pericardium. Confirmed by CA, eleven of thirteen cases with pericardial tamponade (blood volume in the pericardium ranging from 235 to 600 ml) could be detected correctly by means of ultrasound. The definite bleeding source remained undetectable by pmUS [5]. Furthermore, a negative influence of progressive decomposition and associated gas formation on quality of images was described [6]. The latest article in this term, published by Charlier et al., focused on the same correlation between pmUS und CA. Thirty-eight cadavers were investigated including cases of natural and non-natural deaths. In 21 cases, no pathologies at all could be depicted by ultrasound. In the remaining cases, only non-lethal changes were detected, which were partly confirmed by CA [23].

*Conclusion: Only a small amount of studies could be found, using ultrasound in a postmortem setting or to examine superficial lesions. Relevant pathological changes can be depicted. Two studies correlated the ultrasonic results with CA*.

### Minimally invasive autopsy (MIA) supported by ultrasound

A total of 18 MIA publications including the use of ultrasound were published between 1998 and 2020 (Table 2). In eleven studies, the results of MIA and subsequent CA were correlated. One study could be found using EUS. In another study, CT and MRI were performed prior to ultrasound-guided biopsies.

Beginning in 1998, Fariña et al. published the first report on combined ultrasound examination, ultrasound-guided biopsies, and CA. Followed by an investigation with the same setting in 2002, they report a concordance rate between MIA results and CA diagnoses of 92% and 83%, respectively [45, 46]. It remains unclear if in these mentioned publications cases were reported twice or if two totally different cohorts were investigated and described. Limitations are given on cardiovascular diseases and tracheobronchial diseases because alterations like lesions of the cardiac valves or thromboembolism of the pulmonary arteries as well as endoluminal-growing bronchial carcinomas are difficult to depict and to reach for biopsy sampling when investigating from the body surface. A similar approach was performed by the group of Weustink et al. who additionally used CT and MRI followed by ultrasound-guided biopsies to complete the diagnostic statement [44]. Combining these methods, an overall concordance of 77% was reached comparing MIA and CA. Success rates for ultrasound-guided tissue collection were high. As well as in the two aforementioned studies, the detection of cardiac diseases as a cause of death failed. One study could be found performing endoluminal investigations, intraabdominal exploration via a transgastric access, and endoscopic ultrasound (EUS) followed by CA [42]. Eight cases were studied here via EUS, which revealed new pathological findings unknown to the clinicians in three cases (e.g., lymph node and liver metastases). Moreover, in one case, a fine-needle core biopsy of the pancreas could be gained. A systematic comparison of the results with CA has not been performed.

Most of the studies published using ultrasound for tissue sampling were performed suspecting infectious diseases. The group of Cox et al. investigated a cohort of HIV-infected adults by means of MIA and CA [40, 41]. Their first practice study was conducted to compare the success rates of blind needle biopsies vs. ultrasound-guided needle biopsies. They showed that the use of ultrasound significantly increased the success rate for the proper sampling of heart and left kidney. Moreover, ultrasound guidance led to fewer unsuccessful biopsies and could reduce unnecessary efforts. Compared to CA, a complete concordance of 52% could be found for all major diagnoses (infections, malignancies, non-communicable diseases). Interestingly, especially for the detection of tuberculosis, a concordance rate of 79% between MIA and CA was described due to the microbiological and histological investigations of the gained tissues. Yet, no significant increase of concordance rates between blind needle biopsies and ultrasound-guided biopsies could be detected [40]. Another study investigating HIV-positive patients showed high success rates for tissues sampled with high histological and microbiological quality using ultrasound guidance [43]. Infectious diseases were the most common cause of death with Mycobacteria as the leading pathogen.

Beginning in 2013, an ongoing research project called CaDMIA (validation of the minimally invasive autopsy tool for cause of death investigation in developing countries; Maputo, Mozambique; Manaus, Amazonas, Brazil) was established [14]. In this project, an interdisciplinary group of pathologists, microbiologists, epidemiologists, and clinicians focused on the feasibility and validity of MIA in LMICs compared to CA throughout all age groups [33–39]. Although tissue sampling was not performed by ultrasound guidance, a preliminary ultrasonic investigation of the bodies was carried out.

In the given studies, a cause of death determination succeeded in 83 to 100% of cases by MIA (n = 537 in total). Compared to CA, concordance rates of 68 to 90% were reached. Infectious diseases were the leading causes of death. Similar to the studies above, cardiovascular and tracheobronchial diseases were difficult to observe. The group of Hurtado et al. additionally investigated the influence of the PMI (< 24 h vs. > 24 h). For these time intervals, no differences in relevant diagnoses could be detected. However, his study group has not been investigating the influence of a longer PMI and diagnostic accuracy of findings beyond 65 h postmortem. Microbiologically, increasing PMI led to increasing rates of detectable bacteria leading to a potential overestimation of clinical significance of such findings [34].

Another application of MIA using pmUS was shown by the group of Duarte-Neto et al., who investigated deceased during the yellow fever epidemic in Brazil in 2018. They showed high success rates of biopsies for the defined organs (liver, lungs, kidneys, spleen, heart). Histological and virological investigation showed a lethal hepatic failure caused by the virus in 17 of 20 cases. In three cases in which yellow fever could not be confirmed, the cause of death could also be detected as a result of tissue sampling (intraabdominal infection with purulent ascites, pneumonia and pyelonephritis, acute myeloid leukemia). Thus, when compared to CA, a concordance rate of 100% between both methods could be shown determining the cause of death [32].

The latest MIA studies supported by ultrasound were carried out during the ongoing COVID-19 pandemic. Respecting enhanced safety measures, a cohort of ten COVID-19 deceased was examined by the group of Duarte-Neto et al. as well. Representative tissue samples could be acquired reliably with ultrasound guidance for further histological and virological investigation to detect viral RNA [31]. A similar approach was carried out by Brook et al. Except for the kidneys, adequate tissue samples for histological and virological processing could be acquired [30]. One recent study focused on core biopsies of the lungs which could have been taken effectively [29]. A considerably larger cohort (*n* = 30) was investigated compared to the two aforementioned studies within a remarkable short PMI (< 2 h). Tissue of high quality was gained showing patterns of diffuse alveolar damage in nearly all cases.

*Conclusion: High concordance rates between MIA and CA could be confirmed in several studies. In most of the studies, ultrasound is used for tissue sampling, rather than imaging-based diagnostics. Many studies were carried out in LMIC, confirming infectious causes of death*.

### Age estimation using sonography of different bones and their epiphyses

In the time frame from 2008 to 2020, 14 articles were published regarding the ultrasonic description of epiphyseal growth plates of different bones and their correlation to an individual’s age (Table 3) using the minimum age principle. Except for two, all studies refer to four ossification stages of epiphyseal growth plates according to Webb and Suchey (stage 1: no union without separate epiphysis; stage 2: no union with separate epiphysis; stage 3: partial union; stage 4: complete union) [60]. One group investigated the clavicles in cadavers [53]; all other studies were performed in living subjects. In only two studies, results were compared with data gained by CT or MRI [47, 53]. In six studies, the medial clavicle epiphysis was examined which is the preferred region for age estimation as recommended by the German study group on forensic age diagnostics (AGFAD) [61, 62]. The highest number of individuals was examined by Schulz et al. including 616 subjects [56]. They proposed that the occurrence of stage four is associated with an individual that has reached at least 18 years of age meaning full age in Germany. In another study, stage four only occurred in even older individuals with at least 22.9 and 22.5 years for males and females, respectively [19]. Similarly, Quirmbach et al. found stage four in male subjects over 21 years in at least 60% of cases but they also describe six false-positive cases in which stage four was found in younger individuals [59]. Contrary, the group of Gonsior et al. examined 410 individuals and detected stage four on at least one side even in a considerable number younger than 18 years (92/264) [49]. The same group conducted a study including CT and macroscopic examination of cadaveric bones and described an overestimation using sonography in seven of ten cases and propose CT as the remaining gold standard [53]. A similar rating is made by Benito et al. due to an overlapping of stages between age groups [48]. According to the investigation of the medial clavicle epiphysis, the group of Schulz et al. and Schmidt et al. examined also the distal fibular epiphysis, the olecranon, the iliac crest, the distal radius, and the trochanter major of the femur, respectively [51, 52, 54, 55, 57, 58]. Regarding the iliac crest, stage four was detected in females with at least 17.1 years and males with at least 18.0 years. In all other regions, full ossification is reached earlier also compared to the medial clavicle epiphysis, e.g., 13.5 years (females) and 14.5 years (males) for the trochanter major of the femur. Sánchez et al. examined the proximal humeral epiphysis and determined six stages of development [50]. A full fusion is related to an age of at least 17 years in both sexes. In a study very recently published by Herrmann et al., a three-stage grading system comparing ultrasound and MRI-determined evaluation of epiphyseal closure of the knee joint was used. They were able to assign the subjects to different ossification stages by means of ultrasound. Individuals could be determined younger than 18 years when complete closing of the growth plates of the knee joint was not finished yet [47].

*Conclusion: Ultrasonic examination of the epiphyses of different bones can be used to limit the age of a young person. The occurring of ossification stage four in the medial clavicle epiphysis is linked to an age of at least 18 years. In only two studies, the results were compared to CT.*

## Discussion

To the best of our knowledge, this is the very first comprehensive literature review to analyze the applicability of ultrasound in the field of legal medicine. Although this method is commonly used in clinical daily routine, only a few of studies regarding its pre- and postmortem feasibility in forensic cases were published to date.

### Basic conditions concerning (postmortem) ultrasound

To evaluate the potential usefulness of ultrasound in legal medicine, it is necessary to consider which questions should be answered, which information is required as well as which subjects (living individuals vs. cadavers) are investigated. Referring to wounded living patients, only a few studies were published in a forensic context indicating that ultrasound was not in focus of forensic pathologists, probably as the primary treatment is carried out by clinicians. However, since these colleagues may have high expertise in sonography, one could consider adding ultrasound to the documentation process of non-accidental injuries in terms of size, depth, and age of dermal injuries. Potentially, ultrasound may also be able to answer the vitality of dermal injuries. Furthermore, there is an obvious difference between examination of superficial alterations or superficially located organs compared to organs lying in the retroperitoneum. Additionally, one has to differentiate between “common” pathological findings, e.g., bile stones, and primarily potential lethal diagnoses, e.g., pericardial tamponades.

In the cited studies on examination of cadavers, there are comparatively spare statements given on image quality and its potential diagnostic implication. Uchigasaki et al. described a reasonable applicability of ultrasound for the detection of liquids such as ascites, pleural effusion, urine, or pericardial effusion [3–6]. Moreover, the depiction of solid organs such as the liver viewed transcostally is described. Limitations are obvious due to the missing possibility of giving breathing commands, shifting of position, missing verbal description of symptoms, and the inevitable process of decomposition. Especially, the beginning of greenish colorization of the abdominal skin and linked gas buildup in the intestines significantly reduces image quality even according to our own experience with pmUS. In line with that, it is impossible to collect valuable images of highly decomposed bodies limiting the time interval of valuable sonography information to the early postmortem period. For putrefied bodies, especially when searching for osseous injuries, CT scans are of greater value [63]. The PMI in which the pmUS examinations were performed vary from less than 2 h to 192 h [4, 29]. It is proposed that valuable images can be made at least up to 5 days postmortem [4, 6]. In contrast to that, Charlier et al. propose a significantly decreased visibility of structures due to postmortem gas formation, although their cohort was examined even within less than 48 h [23]. Reasons for this broad range of possible examinations within the given PMI may be linked to time between death and beginning of cooling, different causes of death, changing efficiency of ultrasonic devices, and, to a high extent, the investigators’ own experience. Moreover, the temperature of the bodies while on ultrasonic examination seems to play an important role. The colder the body, the harder becomes the subcutaneous fatty tissue and permeation of ultrasound waves decreases tremendously. Uchigasaki et al. propose an examination at the earliest 1 to 2 h after taking the corpse out of the cooling [6]. According to own, to date not yet published experiences, the interval to warm up the corpses should be longer, even up to 8 h because the refrigerated fat seems to significantly reduce image quality. Our practice includes to remove the bodies from the cooling chambers in the night before pmUS is planned. However, if pmUS should be used systematically in the future, the authors argue for a prompt investigation on scene or at the morgue while arrival with a portable device as frequently used in the given studies. This would reduce the PMI to the best possible minimum and could also bypass the problems linked to the cooling of the corpses.

Referring to lethal diagnoses, only a pericardial tamponade was described as a significant finding yet. In their investigation of 455 bodies, Uchigasaki et al. found a pericardial tamponade in eleven of 13 cases when checked by autopsy results [5]. In another study conducted by the same group, forensically relevant findings such as intraabdominal fluid (ascites or bleeding), pleural effusions, or metastases could be detected in 25% of the investigated 158 cases [4]. However, it has not been mentioned, how often such diagnoses were existing in the full cohort of cases. In contrast to that, Charlier et al. examining 38 cadavers could only find non-lethal diagnoses and missed the cause of death in any case of natural and non-natural deaths [23]. Concerning non-natural deaths, the abovementioned studies failed to depict relevant findings causing death like in drowning, hanging, or concerning lethal injuries. Thus, these results indicate limitations of ultrasound in the wide range of main diagnoses of forensic case work (Table 4).

**Table 4:** Advantages and disadvantages of sonography in a forensic context

Advantages	Disadvantages
High flexibility and availability	Lower image quality compared to computed tomography or magnetic resonance imaging
Inexpensiveness	Biased by investigator’s experience
Not tied to a certain place	Limitations due to putrefaction / cooling
Increased success rate for tissue sampling compared to blind biopsies	Limitations for cerebral, cardiovascular, and tracheobronchial pathologies
Examination of superficial lesions	Limited experience regarding non-natural causes of death
No radiation	
Higher acceptance rate in population (to be proven)	

Similar to clinical settings, imaging techniques are linked to a question dealing with symptoms the patient indicates. This information offers relevant hints to the physician about the potential underlying disease and to plan further diagnostics. The same principle can be applied in legal medicine especially when no CA and thus no explicit inspection of organs is performed. Knowing pre-existing illnesses as well as symptoms and circumstances prior to death can valuably support the ultrasonic examination of the deceased. For example, if a chronic heart failure due to a coronary artery disease was known, the leading symptom prior to death was progressive dyspnea and the ultrasonic examination reveals pleural effusions and signs of an interstitial edema, the cause of death can potentially be attributed to a cardiac decompensation with signs of acute left heart failure.

In this form, the sole ultrasonic investigation could be seen as a potential extension or one step further regarding the internationally acknowledged tool of verbal autopsy (World Health Organization) when an autoptic investigation of the corpse is not allowed by next of kin [64]. Therefore, adding imaging impressions from the inside of a body could enhance the informative value when compared to a sole external postmortem investigation. Especially in countries with low infrastructures and healthcare systems where many deaths occur unregistered and without proper death certificates, verbal autopsies contribute to rank cause-specific mortalities [65–67].

The informative value of ultrasound examinations is of course significantly depending on the examiners’ skills. For clinicians, a standardized education including training programs, defined numbers of executed examinations, and at last daily practice under supervision is essential for qualified examinations in routine and emergency diagnostics [68–71]. Based on the different curricula, most forensic pathologists were not educated in using ultrasound, whereas postmortem CT diagnostics found its way in the forensic practice and routine [9]. Only few of the authors cited in this review explicitly declared that either the ultrasonic examinations or the tissue sampling was carried out by trained staff, e.g., radiologists [23, 32, 44]. In nearly all studies on age estimation, the examinations were performed by either qualified arthrosonographists or the physicians prepared by attending the introductory course for the locomotor system by the DEGUM (German Society for Ultrasound in Medicine) [19, 49, 53–56, 58, 59]. Many authors state a learning curve for time and execution of the investigations, success rate of organ punctures, and quality of gained tissue samples which goes in line with the aforementioned necessity of training. Moreover, the postmortem conditions differ to patients alive so investigators experienced in clinical examinations need to adjust to these variations.

*Conclusion: Postmortem ultrasonic investigations are limited due to the postmortem changes of the bodies. The detection of lethal diagnoses by pure imaging is infrequently successful. Ultrasonic examinations of living individuals are rarely performed by forensic pathologists. Interdisciplinary education is necessary and recommended.*

### The minimally invasive approach

A promising field in postmortem investigations was established regarding MIAs. For many decades, collecting of tissue samples using biopsy needles and subsequent histological examinations to specify pathologies have been practiced [72–75]. The samples were gathered without any imaging techniques only by using anatomical landmarks. Especially regarding organs difficult to reach, such as the kidneys, success rates yielded limited outcomes. Foroudi et al. successfully biopsied the kidneys in only 9.5% of 21 cases and Wellmann described success rates for the kidneys of 33.6% in 394 cases [72, 75]. Success rates of other organs varied from 45.7 (lungs) to 100% (liver and heart) [73, 75]. Huston et al. gained a concordance rate of 67% for determination of cause of death compared to CA, whereas Foroudi et al. only reached 43% [72, 73]. At the end of the 1990s, Fariña et al. introduced ultrasound to postmortem investigations and showed considerably achievements of improving biopsy taking [45, 46]. Retroperitoneal located organs like kidneys or spleen were successfully biopsied in all cases as well as the lungs. Only the heart was reached in 92% of the 100 cases [45].

Concerning MIA, in most of the studies included in this review, ultrasound was rather a supportive tool for gaining the tissue samples than for diagnostic imaging purposes. Excluding a few studies, the examined cohorts were either highly infectious deceased or populations in LMICs. Regarding the latter, the investigations were considerably promoted by the CaDMIA project [14, 16, 33–39]. The project is based in countries where healthcare structures do not allow frequent CA and thus missing important information on causes of death to improve the healthcare systems. Reasons for that are missing cultural acceptance of complete opening of the body, a lack of understanding for the purpose of the examinations, missing educated staff or logistic aspects like cooling, and transportation of the deceased [15, 76, 77]. Due to more flexible and barely disfiguring examination options, MIA offers the possibility to increase cause of death determination. In the studies cited, it was shown that after ultrasonic evaluation of organs and body fluids, usable tissue samples could be gained. Finally, overall concordance rates between MIA and CA of 68 to 90% were reached. Furthermore, investigations on acute infections like yellow fever or the recent SARS-CoV-2 pandemic showed reasonable applicability for ultrasound-supported tissue sampling for further diagnostics and cause of death determination [29–32]. Additionally, concerning biosafety and protection of medical staff, needle biopsies supported by ultrasound are possible without opening of the corpse and thus reducing the infection risks for the investigators themselves to a minimum.

The high concordance rates given in the MIA studies seem promising but most of cases died due to acute or underlying severe chronic infectious diseases like HIV or tuberculosis. Keeping this in mind, examinations of deceased in HIC who frequently suffer from so-called lifestyle diseases as well as sudden cardiac deaths or intracranial pathologies require another perspective on the gained tissues and images [78, 79]. This goes in line with previous described limited accessibility of cardiac and tracheobronchial tissue samples [44, 45]. Next to this, no pmUS or MIA approaches for intracranial lesions or for sampling of cerebrospinal fluid have been evaluated yet but are already planned by our working groups.

Regarding these anatomical regions, ultrasound is strictly limited due to air formation, either when there are air bubbles in the atria or ventricles of the heart as a result of decomposition or even gas embolisms or simply when the lungs cover the heart, which makes it nearly impossible to depict the heart. In such cases, CT or MRI offer valuable information and the opportunity to detect coronary artery sclerosis and relevant stenoses, pulmonary embolism, and inflammatory alterations like myocarditis or arrhythmogenic structural alterations [80, 81]. Concerning given concordance rates obtained by minimally invasive methods combining cross-sectional imaging, added angiography and tissue sampling higher values were reached. For example, Ross et al. could detect the cause of death in 19 of 20 cases with postmortem CT angiography and CT-guided biopsies compared to subsequent CA focusing on sudden deaths after chest pain [82]. Similar values were reached by Bolliger et al., who report identical results in 18 of 20 cases [83]. Blokker et al. and Westphal et al. report an agreement on cause of death determined by MIA and CA in 92% and 80% of cases, respectively, in which the investigations of Westphal et al. did not include histological investigations [84, 85].

In these cases, quoted concordance rates are only partly comparable as the examined cohorts partially widely differ in underlying diseases and socio-economic origin, e.g., the aforementioned severe infectious diseases vs. coronary artery disease in western countries. Moreover, the applied imaging methods of CT and MRI offer a significantly better image quality than ultrasound, especially for the heart and lungs as well as for intracerebral lesions. The addition of angiography allows diagnostic imaging for small vessels like the coronary arteries which are nearly impossible to depict with ultrasound [86]. Although the cross-sectional techniques are truly beneficial, they are more elaborative and more expensive than ultrasonic diagnostics. Additionally, they are linked to a specific place where the devices are located, are rarely available in forensic institutes and morgues, and need specialized trained staff to be operated. This point shows that MIA with CT and MRI is less flexible and highly dependent on socio-economic and healthcare structures of countries. Although no explicit charges are available, ultrasonic investigations seem obviously cheaper. Furthermore, investigators are more flexible where to perform the examinations, in particular when portable ultrasonic devices are used. This allows, among other things, examinations in a distinct short PMI (commonly less than 2 h) [29].

In the previous paragraphs, some limitations on diagnoses possible to detect by ultrasonic examinations were already given. In this line, needed to be mentioned are cerebral hemorrhages, intraabdominal lesions and operations, e.g., resections of parts of the bowel, as well as septic diseases when there is no septic focus detectable by means of ultrasound. In (attempted) homicides or suicides, ultrasound could only partly provide further information, e.g., when there is free fluid due to organ injuries or in describing the depth of superficial wounds [24, 28]. Lesions occurring in cases of compressive violence against the neck like fractures of the hyoid bone can potentially be detected by CT but seem theoretically difficult to depict by ultrasound [87].

*Conclusion: Ultrasound improves the success rates of biopsies. Tissue samples for further histopathological and microbiological investigations can be accurately gathered. There still remain limitations for cardiovascular, intracerebral, and non-natural causes of death*.

### Ultrasound as a method for forensic age estimation without radiation exposure?

Apart from postmortem or injury assessment settings, ultrasound has the potential to play an important role in forensic age estimation. Current practice as recommended by the AGFAD, examining individuals includes physical examination (body data, maturity signs, hints for development disorders), dental examination, and radiography of the hand to depict maturation of the skeletal system. If the bones of the hands are completely fused already, examination of the medial clavicle epiphysis by means of CT or MRI is recommended [18, 61, 62]. Radiation doses for the exposed are calculated low for the specific area (e.g., 0.4 mSv for CT, covering a distance of 10 cm of the upper thorax). Basically, examinations are not evaluated as a health hazard or disadvantage for the examined young(er) people [62, 88]. Nevertheless, it must be accounted as an additive radiation exposure, additional to the naturally occurring background radiation. Thus, not all European countries follow these instructions, in order to protect minors [48]. Moreover, device distribution widely differs among countries [89]. Concerning ongoing migration movements via different migration routes to mainly Europe, many countries have to deal with daily arriving people who need to be properly identified [48, 90]. For these purposes, ultrasound could offer a flexible, cheap, and easy to handle alternative to examine alleged minors concerning their age. Many epiphyseal growth plates of different bones were examined by means of ultrasound like the iliac crest, the olecranon, or the radius, but most often, the medial clavicle epiphysis was investigated most probable because of the already established knowledge on this epiphyseal region using CT or MRI. The authors describe a secure applicability and depiction of the epiphyses and most authors assign the occurrence of ossification stage four to an age over 18 years, whereas a clear differentiation between 18 and 21 years was yet impossible using the four-stage system [19, 48, 56, 59]. In contrast to that, the group of Gonsior et al. found a considerable number of individuals younger than 18 years with fully ossified epiphyses some being not older than 14 years [49]. These results show one crucial aspect of ultrasound, which was described above already, namely the dependence of investigator. To perform the examinations, ultrasound skills need to be trained and a coherent education on the sono-morphological aspects is needed. Only one study could be found, comparing the ultrasonic results with the macroscopic aspects during autopsy. In this study, ten medial clavicle epiphyses were examined using both methods, and in seven of ten cases, results differed due to overestimation of the age by ultrasound [53]. Compared to all other cited studies on age estimation, this study had the lowest case numbers (five individuals, investigation bilaterally), whereas other groups investigated up to 616 people [56]. Overestimation by ultrasound is mainly ascribed to anatomical variations of the bones or the depth of the surrounding soft tissue. Additionally, it is conceivable that physical access of the ultrasonic probe to the epiphysis is limited due to each individual’s anatomy. These factors determine image quality and the resulting assessment and are overcome easier by CT.

Regarding the aforementioned efforts, ultrasound has the potential to become an established tool in forensic age estimation. However, further research and comparative studies on the concordance of ultrasonic results and CT are necessary. In addition, standardized procedures and coherent education of forensic physicians using ultrasound needs to be established.

*Conclusion: The examination of the medial clavicle epiphysis is the region preferred for forensic age estimation. Ultrasound offers the possibility of cheap and flexible examinations, without the need for radiation exposure. Yet, further correlation studies between ultrasonic and CT results are needed.*

## Future directions

In response to decreasing autopsy rates, the offer of well-proven imaging and minimally invasive techniques could help to gain better acceptance of necessary and important postmortem examinations in all parts of the world. During the lately occuring COVID-19 pandemic, the need for quick, well-founded, and secure research was inevitable clear more than once using postmortem results and tissues [91, 92]. To reduce the risk of infections of medical staff in autopsies on deceased carrying highly contagious diseases, ultrasound techniques can be a valuable tool to gain relevant findings without complete opening of the corpses and can help to classify deceased based on sono-morphological patterns like developed for clinical patients [93, 94]. Moreover, ultrasound can be used to guide all diagnostic relevant tissue samples as well as bodily fluids like urine, pleural effusions, or ascites for histopathological and microbiological examination. Especially in infectious causes of death, a protocolized process of investigation helps to detect relevant pathogens and their potential lethal meaning [95]. Concerning this, MIA supported by ultrasound could evolve to a widely used method in both forensic and clinical pathology helping to understand newly emerging diseases both in HIC and in countries where medical structures are limited. Moreover, routine cases could be investigated, when a CA is impossible due to several and already mentioned reasons. To date, no general education of forensic pathologists in using ultrasound takes place and ultrasonic application is mostly reserved to clinical routine. As interdisciplinarity plays a key role in modern medicine and ultrasonic examinations deserve experienced investigators, collective trainings could be stimulated to improve diagnostic abilities on both sides [96].

## Conclusion

Ultrasound has the potential to valuably support forensic pathologists in pre- and postmortem conditions. As for any other investigation given, limitations of ultrasound should be strictly considered. Certainly, conventional autopsies (inclusive CT, histopathological, microbiological, or laboratory diagnostics, where required) will inevitably remain the gold standard in many aspects. Nevertheless, non- or minimally invasive methods can provide valid results with increasing accuracy, could lead to a better acceptance of postmortem investigations, and thus update the current gold standard. Both for solely imaging and for gathering of tissue samples for further histological or laboratory-based diagnostics ultrasound is a widely available, easy to handle and comparatively cheap tool which can be operated worldwide without being tied to a certain place. Due to the comparatively limited count of studies using ultrasound in the field of legal medicine, more extensive validation studies are essential to finally define the most adequate implementation of this modality in a pre- and postmortem setting. There is need for further investigation of ultrasonic feasibility in non-natural causes of death, adjustments to mortality cohorts in high-income countries, especially regarding cardiovascular pathologies, as well as applicability in age estimation or forensic examinations of living victims of violence.

## Data Availability

Requests for materials should be addressed to D. M. or B. O.

## References

[1] Quan L, Zhu BL, Fujita MQ, Maeda H (2003). Ultrasonographic densitometry of the lungs at autopsy: a preliminary investigation for possible application in forensic pathology. Legal Med.

[2] Webb PA, Terry HJ, Gee DJ (1986). A method for time of death determination using ultrasound—a preliminary report. J Forensic Sci Soc.

[3] Uchigasaki S, Lach H, Oesterhelweg L, Sperhake JP, Püschel K, Oshida S (2003). Postmortale Urinmengenmessung mit Ultraschall. Rechtsmedizin.

[4] Uchigasaki S, Oesterhelweg L, Gehl A (2004). Application of compact ultrasound imaging device to postmortem diagnosis. Forensic Sci Int.

[5] Uchigasaki S, Oesterhelweg L, Sperhake JP, Püschel K, Oshida S (2006). Application of ultrasonography to postmortem examination. Diagnosis of pericardial tamponade. Forensic Sci Int.

[6] Uchigasaki S (2006) Postmortem ultrasound imaging in forensic pathology. In: Tsokos M (ed) Forensic pathology reviews, vol 4. Forensic Pathology Reviews. Humana Press 405–412. 10.1007/978-1-59259-921-9_13

[7] McLane HC, Berkowitz AL, Patenaude BN (2015). Availability, accessibility, and affordability of neurodiagnostic tests in 37 countries. Neurology.

[8] Ngoya PS, Muhogora WE, Pitcher RD (2016) Defining the diagnostic divide: an analysis of registered radiological equipment resources in a low-income African country. Pan Afr Med J 25:99. 10.11604/pamj.2016.25.99.9736 10.11604/pamj.2016.25.99.9736 PMC5325496 28292062

[9] Blokker BM, Wagensveld IM, Weustink AC, Oosterhuis JW, Hunink MGM (2015). Non-invasive or minimally invasive autopsy compared to conventional autopsy of suspected natural deaths in adults: a systematic review. Eur Radiol.

[10] Blokker BM, Weustink AC, Hunink MGM, Oosterhuis JW (2017). Autopsy rates in the Netherlands: 35 years of decline. Terry J, ed. PLoS ONE.

[11] Kuijpers CCHJ, Fronczek J, van de Goot FRW, Niessen HWM, van Diest PJ, Jiwa M (2014). The value of autopsies in the era of high-tech medicine: discrepant findings persist. J Clin Pathol.

[12] Shojania KG, Burton EC (2008). The vanishing nonforensic autopsy. N Engl J Med.

[13] Wagensveld IM, Weustink AC, Kors JA, Blokker BM, Hunink MGM, Oosterhuis JW (2020). Effect of minimally invasive autopsy and ethnic background on acceptance of clinical postmortem investigation in adults. Holda MK, ed. PLoS ONE.

[14] Bassat Q, Ordi J, Vila J (2013). Development of a post-mortem procedure to reduce the uncertainty regarding causes of death in developing countries. Lancet Glob Health.

[15] Bassat Q, Castillo P, Alonso PL, Ordi J, Menéndez C (2016). Resuscitating the dying autopsy. PLoS Med.

[16] Bassat Q (2017). Minimally invasive autopsy: welcoming a new tool for cause of death investigation in children in resource-constrained countries. J Trop Pediatr.

[17] Byass P (2016). Minimally invasive autopsy: a new paradigm for understanding global health?. PLoS Med.

[18] Buckley MB, Clark KR (2017). Forensic age estimation using the medial clavicular epiphysis: a study review. Radiol Technol.

[19] Schulz R, Zwiesigk P, Schiborr M, Schmidt S, Schmeling A (2008). Ultrasound studies on the time course of clavicular ossification. Int J Legal Med.

[20] Wohlin C (2014) Guidelines for snowballing in systematic literature studies and a replication in software engineering. In: Proceedings of the 18th International Conference on Evaluation and Assessment in Software Engineering - EASE ’14. ACM Press 1–10. 10.1145/2601248.2601268

[21] Ichioka H, Miyamori D, Ishikawa N (2020). Estimation of cadaveric age by ultrasonography. Diagnostics (Basel).

[22] Helm T, Bir C, Chilstrom M, Claudius I (2016). Ultrasound characteristics of bruises and their correlation to cutaneous appearance. Forensic Sci Int.

[23] Charlier P, Chaillot P-F, Watier L (2013). Is post-mortem ultrasonography a useful tool for forensic purposes?. Med Sci Law.

[24] Mimasaka S, Oshima T, Ohtani M (2012). Characterization of bruises using ultrasonography for potential application in diagnosis of child abuse. Leg Med (Tokyo).

[25] Bhutani MS, Arantes VN, Verma D, Moezzi J (2009). Histopathologic correlation of endoscopic ultrasound findings of chronic pancreatitis in human autopsies. Pancreas.

[26] Seifert D, Püschel K (2006). Subgaleal hematoma in child abuse. Forensic Sci Int.

[27] Cotton JM, Cooke JC, Monaghan MJ (2000). Forensic echocardiography: a case in point. Echocardiography.

[28] Gniadecka M, Danielsen L (1995). High-frequency ultrasound for torture-inflicted skin lesions. Acta Derm Venereol (Stockh).

[29] Li Y, Wu J, W S,  (2021). Progression to fibrosing diffuse alveolar damage in a series of 30 minimally invasive autopsies with COVID-19 pneumonia in Wuhan, China. Histopathology.

[30] Brook OR, Piper KG, Mercado NB (2020). Feasibility and safety of ultrasound-guided minimally invasive autopsy in COVID-19 patients. Abdom Radiol (NY).

[31] Duarte-Neto AN, Monteiro RAA, Silva LFF (2020). Pulmonary and systemic involvement in COVID-19 patients assessed with ultrasound-guided minimally invasive autopsy. Histopathology.

[32] Duarte-Neto AN, de Monteiro RA, A, Johnsson J,  (2019). Ultrasound-guided minimally invasive autopsy as a tool for rapid post-mortem diagnosis in the 2018 Sao Paulo yellow fever epidemic: correlation with conventional autopsy. Petersen CA, ed. PLoS Negl Trop Dis.

[33] Palhares AEM, Ferreira L, Freire M (2019). Performance of the minimally invasive autopsy tool for cause of death determination in adult deaths from the Brazilian Amazon: an observational study. Virchows Arch.

[34] Hurtado JC, Quintó L, Castillo P (2018). Postmortem interval and diagnostic performance of the autopsy methods. Sci Rep.

[35] Bassat Q, Castillo P, Martínez MJ (2017). Validity of a minimally invasive autopsy tool for cause of death determination in pediatric deaths in Mozambique: an observational study. Byass P, ed. PLoS Med.

[36] Castillo P, Hurtado JC, Martínez MJ (2017). Validity of a minimally invasive autopsy for cause of death determination in maternal deaths in Mozambique: an observational study. Byass P, ed. PLoS Med.

[37] Castillo P, Martínez MJ, Ussene E (2016). Validity of a minimally invasive autopsy for cause of death determination in adults in Mozambique: an observational study. Byass P, ed. PLoS Med.

[38] Castillo P, Ussene E, Ismail MR (2015). Pathological methods applied to the investigation of causes of death in developing countries: minimally invasive autopsy approach. Cappello F, ed. PLoS ONE.

[39] Martínez MJ, Massora S, Mandomando I (2016). Infectious cause of death determination using minimally invasive autopsies in developing countries. Diagn Microbiol Infect Dis.

[40] Cox JA, Lukande RL, Kalungi S (2014). Needle autopsy to establish the cause of death in HIV-infected hospitalized adults in Uganda: a comparison to complete autopsy. J Acquir Immune Defic Syndr.

[41] Cox JA, Lukande RL, Kalungi S (2014). Practice of percutaneous needle autopsy; a descriptive study reporting experiences from Uganda. BMC Clin Pathol.

[42] Denzer UW, von Renteln D, Lübke A (2013). Minimally invasive autopsy by using postmortem endoluminal and transluminal endoscopy and EUS. Gastrointest Endosc.

[43] Wong EB, Omar T, Setlhako GJ (2012). Causes of death on antiretroviral therapy: a post-mortem study from South Africa. Kranzer K, ed. PLoS ONE.

[44] Weustink AC, Hunink MGM, van Dijke CF, Renken NS, Krestin GP, Oosterhuis JW (2009). Minimally invasive autopsy: an alternative to conventional autopsy?. Radiology.

[45] Fariña J, Millana C, Fdez-Aceñero JM (2002). Ultrasonographic autopsy (echopsy): a new autopsy technique. Virchows Arch.

[46] Fariña J, Millana C (1998). Applications of ultrasonography on the post-mortem examination (ecopsy) in humans. J Echogr Med Ultrasons.

[47] Herrmann J, Säring D, Auf der Mauer M, Groth M, Jopp-van Well E (2020). Forensic age assessment of the knee: proposal of a new classification system using two-dimensional ultrasound volumes and comparison to MRI. Eur Radiol.

[48] Benito M, Muñoz A, Beltrán I, Labajo E, Perea B, Sánchez JA (2018). Assessment of adulthood in the living Spanish population based on ossification of the medial clavicle epiphysis using ultrasound methods. Forensic Sci Int.

[49] Gonsior M, Ramsthaler F, Birngruber C, Obert M, Verhoff MA (2016). The completely fused medial clavicular epiphysis in high-frequency ultrasound scans as a diagnostic criterion for forensic age estimations in the living. Int J Legal Med.

[50] Sánchez MB, Codinha S, García AM, Sánchez JAS (2017). Estimating legal age based on fusion of the proximal humeral epiphysis. Int J Legal Med.

[51] Schmidt S, Schiborr M, Pfeiffer H, Schmeling A, Schulz R (2014). Ossifikationsvorgänge des Trochanter major femoris: Bedeutung für die forensische Altersschätzungspraxis Lebender. Rechtsmedizin.

[52] Schulz R, Schiborr M, Pfeiffer H, Schmidt S, Schmeling A (2014). Forensic age estimation in living subjects based on ultrasound examination of the ossification of the olecranon. J Forensic Leg Med.

[53] Gonsior M, Ramsthaler F, Gehl A, Verhoff MA (2013). Morphology as a cause for different classification of the ossification stage of the medial clavicular epiphysis by ultrasound, computed tomography, and macroscopy. Int J Legal Med.

[54] Schmidt S, Schiborr M, Pfeiffer H, Schmeling A, Schulz R (2013). Age dependence of epiphyseal ossification of the distal radius in ultrasound diagnostics. Int J Legal Med.

[55] Schmidt S, Schiborr M, Pfeiffer H, Schmeling A, Schulz R (2013). Sonographic examination of the apophysis of the iliac crest for forensic age estimation in living persons. Sci Justice.

[56] Schulz R, Schiborr M, Pfeiffer H, Schmidt S, Schmeling A (2013). Sonographic assessment of the ossification of the medial clavicular epiphysis in 616 individuals. Forensic Sci Med Pathol.

[57] Schulz R, Schiborr M, Pfeiffer H, Schmidt S, Schmeling A (2013). Sonographic examination on the time frame of ossification of the distal fibula epiphysis. Arch Kriminol.

[58] Schmidt S, Schmeling A, Zwiesigk P, Pfeiffer H, Schulz R (2011). Sonographic evaluation of apophyseal ossification of the iliac crest in forensic age diagnostics in living individuals. Int J Legal Med.

[59] Quirmbach F, Ramsthaler F, Verhoff MA (2009). Evaluation of the ossification of the medial clavicular epiphysis with a digital ultrasonic system to determine the age threshold of 21 years. Int J Legal Med.

[60] Webb PA, Suchey JM (1985). Epiphyseal union of the anterior iliac crest and medial clavicle in a modern multiracial sample of American males and females. Am J Phys Anthropol.

[61] Arbeitsgemeinschaft für Forensische Altersdiagnostik. (AGFAD). https://www.medizin.uni-muenster.de/en/rechtsmedizin/schmeling/agfad/about/home. Last accessed on 25 January 2021

[62] Schmeling A, Dettmeyer R, Rudolf E, Vieth V, Geserick G (2016). Forensic age estimation—methods, certainty, and the law. Dtsch Arztebl Int.

[63] Thali MJ, Yen K, Schweitzer W, Vock P, Ozdoba C, Dirnhofer R (2003). Into the decomposed body—forensic digital autopsy using multislice-computed tomography. Forensic Sci Int.

[64] World Health Organization (2016) Verbal autopsy standards: the 2016 WHO verbal autopsy instrument. WHO, Geneva. https://www.who.int/healthinfo/statistics/verbalautopsystandards/en/. Last accessed on 25 January 2021

[65] Byass P, Herbst K, Fottrell E (2015). Comparing verbal autopsy cause of death findings as determined by physician coding and probabalistic modelling: a public health analysis of 54 000 deaths in Africa and Asia. J Glob Health.

[66] Dehghan A, Nasirian M, Haghdoost AA, Bahramali E, Sharifi H (2018) Validation of the verbal autopsy questionnaire for adult deaths in Iran. Med J Islam Republic Iran 32(1):33–36. 10.14196/mjiri.32.7 10.14196/mjiri.32.7 PMC6108282 30159258

[67] Polprasert W, Rao C, Adair T, Pattaraarchachai J, Porapakkham Y, Lopez AD (2010). Cause-of-death ascertainment for deaths that occur outside hospitals in Thailand: application of verbal autopsy methods. Popul Health Metrics.

[68] Bhagra A, Tierney DM, Sekiguchi H, Soni NJ (2016). Point-of-care ultrasonography for primary care physicians and general internists. Mayo Clin Proc.

[69] Nicholls D, Sweet L, Hyett J (2014). Psychomotor skills in medical ultrasound imaging: an analysis of the core skill set. J Ultrasound Med.

[70] Østergaard ML, Rue Nielsen K, Albrecht-Beste E, Kjær Ersbøll A, Konge L, Bachmann Nielsen M (2019). Simulator training improves ultrasound scanning performance on patients: a randomized controlled trial. Eur Radiol.

[71] Valentin L (2006). Minimum training recommendations for the practice of medical ultrasound. European Federation of Societies for Ultrasound in Medicine and Biology. Ultraschall Med.

[72] Foroudi F, Cheung K, Duflou J (1995). A comparison of the needle biopsy post mortem with the conventional autopsy. Pathology.

[73] Huston BM, Malouf NN, Azar HA (1996). Percutaneous needle autopsy sampling. Mod Pathol.

[74] Terry R (1955). Needle necropsy. J Clin Pathol.

[75] Wellmann KF (1969). The needle autopsy: a retrospective evaluation of 394 consecutive cases. Am J Clin Pathol.

[76] Bunei M, Muturi P, Otiato F (2019). Factors influencing acceptance of post-mortem examination of children at a tertiary care hospital in Nairobi, Kenya. Ann Glob Health.

[77] Lishimpi K (2001). Necropsies in African children: consent dilemmas for parents and guardians. Arch Dis Childhood.

[78] Gaziano TA (2017). Lifestyle and cardiovascular disease: more work to do. J Am Coll Cardiol.

[79] Scholz M, Henger S, Beutner F (2020). Cohort profile: the Leipzig Research Center for Civilization Diseases-Heart Study (LIFE-Heart). Int J Epidemiol.

[80] Wichmann D, Heinemann A, Weinberg C (2014). Virtual autopsy with multiphase postmortem computed tomographic angiography versus traditional medical autopsy to investigate unexpected deaths of hospitalized patients: a cohort study. Ann Intern Med.

[81] Puranik R, Gray B, Lackey H (2014). Comparison of conventional autopsy and magnetic resonance imaging in determining the cause of sudden death in the young. J Cardiovasc Magn Reson.

[82] Ross SG, Thali MJ, Bolliger S, Germerott T, Ruder TD, Flach PM (2012). Sudden death after chest pain: feasibility of virtual autopsy with postmortem CT angiography and biopsy. Radiology.

[83] Bolliger SA, Filograna L, Spendlove D, Thali MJ, Dirnhofer S, Ross S (2010). Postmortem imaging-guided biopsy as an adjuvant to minimally invasive autopsy with CT and postmortem angiography: a feasibility study. Am J Roentgenol.

[84] Blokker BM, Weustink AC, Wagensveld IM (2018). Conventional autopsy versus minimally invasive autopsy with postmortem MRI, CT, and CT-guided biopsy: comparison of diagnostic performance. Radiology.

[85] Westphal SE, Apitzsch JC, Penzkofer T, Kuhl CK, Mahnken AH, Knüchel R (2014). Contrast-enhanced postmortem computed tomography in clinical pathology: enhanced value of 20 clinical autopsies. Hum Pathol.

[86] Grabherr S, Heinemann A, Vogel H (2018). Postmortem CT angiography compared with autopsy: a forensic multicenter study. Radiology.

[87] Gascho D, Heimer J, Tappero C, Schaerli S (2019). Relevant findings on postmortem CT and postmortem MRI in hanging, ligature strangulation and manual strangulation and their additional value compared to autopsy – a systematic review. Forensic Sci Med Pathol.

[88] Meier N, Schmeling A, Losse R, Vleth V (2015). Altersdiagnostik und Strahlenexposition. Rechtsmedizin.

[89] Statista. https://de.statista.com/statistik/daten/studie/182666/umfrage/computertomographen-anzahl-in-europa/. Last accessed on 24 Jan 2021

[90] European Agency for the Management of Operational Cooperation at the External Borders of the Member States of the European Union (Frontex) (2021). https://frontex.europa.eu/along-eu-borders/migratory-map/. Last accessed 24 January 2021

[91] Edler C, Schröder AS, Aepfelbacher M (2020). Dying with SARS-CoV-2 infection—an autopsy study of the first consecutive 80 cases in Hamburg, Germany. Int J Legal Med.

[92] Sperhake J-P (2020). Autopsies of COVID-19 deceased? Absolutely!. Leg Med.

[93] Kiefl D, Eisenmann S, Michels G (2020). Empfehlungen zur Lungen- und Thoraxsonographie bei Patienten mit COVID-19-Erkrankung. Med Klin Intensivmed Notfmed.

[94] Kanchan T, Shrestha R, Krishan K (2020). Post-mortem ultrasonography: a safer alternative to autopsies in COVID-19 deaths. J Ultrasound.

[95] Saegeman V, Cohen MC, Burton JL (2021). Microbiology in minimally invasive autopsy: best techniques to detect infection. ESGFOR (ESCMID study group of forensic and post-mortem microbiology) guidelines. Forensic Sci Med Pathol.

[96] Püschel K, Heinemann A, Dietz E, Hellwinkel O, Henners D, Fitzek A (2020). New developments and possibilities in the field of post-mortem medicine mortui vivos docent. Rechtsmedizin.

